# Exploring toilet plume bioaerosol exposure dynamics in public toilets using a Design of Experiments approach

**DOI:** 10.1038/s41598-024-61039-w

**Published:** 2024-05-09

**Authors:** Elizabeth N. Paddy, Oluwasola O. D. Afolabi, M. Sohail

**Affiliations:** School of Architecture, Building and Civil Engineering, Loughborough University, Loughborough, Leicestershire UK

**Keywords:** Environmental sciences, Pathogens, Policy and public health in microbiology, Civil engineering

## Abstract

Bioaerosols generated during toilet flushing can contribute to the spread of airborne pathogens and cross-contamination in indoor environments. This presents an increased risk of fomite-mediated or aerosol disease transmission. This study systematically investigated the factors contributing to increased bioaerosol exposure following toilet flushing and developed an empirical model for predicting the exposure-relevant bioaerosol concentration. Air in a toilet cubicle was sampled by impaction after seeding with *Clostridium difficile* spores. Design of Experiments (DoE) main effects screening and full factorial design approaches were then employed to investigate the significant factors that heighten the risk of exposure to bioaerosols post-flush. Our findings reveal that the inoculated bacterial concentration (*C*), time elapsed after flushing (*t*), lateral distance (*d*), and mechanical ventilation (*v*) are significant predictors of bioaerosol concentration, with p-values < 0.05. The interaction term, *C* × *d* showed a marked increase in bioaerosol concentration up to 232 CFU/m^3^ at the closest proximity and highest pathogen load. The interplay of C and t (*C* × *t*) demonstrated a time-dependent attenuation of bioaerosol viability, with concentrations peaking at 241 CFU/m^3^ immediately post-flush and notably diminishing over time. The lateral distance and time post-flush (d × t) interaction also revealed a gradual decrease in bioaerosol concentration, highlighting the effectiveness of spatial and temporal dilution in mitigating bioaerosol exposure risks. Furthermore, there is an immediate rise in relative humidity levels post-flush, impacting the air quality in the toilet environment. This study not only advances our understanding of exposure pathways in determining bioaerosol exposure, but also offers pivotal insights for designing targeted interventions to reduce bioaerosol exposure. Recommendations include designing public toilets with antimicrobial surfaces, optimizing ventilation, and initiating timely disinfection protocols to prioritise surfaces closest to the toilet bowl during peak exposure periods, thereby promoting healthier indoor environments and safeguarding public health in high-traffic toilet settings.

## Introduction

Toilet flushing generates infectious bioaerosols from urine, faeces, or vomit shed into a toilet bowl^1–5^. These bioaerosols either settle on surfaces in the washroom or remain airborne, presenting an increased risk of fomite-mediated or aerosol disease transmission^6–8^. The risk of aerosol disease transmission has gained much attention ever since toilet plume was implied as the source of a 187-person Severe Acute Respiratory Syndrome (SARS) outbreak^9^ and pathogens such as *Staphylococcus aureus* and *Legionella* sp., responsible for respiratory infections were isolated in toilet plume^4,10–12^. Studies have classified toilet plume bioaerosols to range between 0.3 to 3 µm in size and capable of reaching heights of about 1.5 m from the ground^13^. This reflects their ability to be inhaled and deposited into the respiratory tract^14^ leading to respiratory infections and emphasising the urgent need for effective interventions.

Adherence to effective handwashing practices and the implementation of appropriate cleaning protocols have significantly decreased the likelihood of surface contamination and the consequent risk of fomite-mediated disease transmission by bioaerosols^7,15,16^. It is important to recognise that even though it may be true that some people practice good hygiene, not everyone follows proper hygiene practices consistently. A 2015 study reveals that only 26.2% of global toilet visits with potential faecal contact were followed by hand washing with soap^17^. Furthermore, in environments where there is a high health risk, such as healthcare settings (including hospitals, medical centres, care homes, and other institutions that care for patients), the larger number of individuals with underlying health conditions and weakened immune systems increases the vulnerability to Healthcare-Acquired Infections (HAIs), which is associated with a higher risk of mortality^18^. Consequently, exposure to infectious toilet plume bioaerosols due to flushing faeces from patients can increase the chances of having multiple HAIs and the development of antimicrobial resistance^19^ in other immunocompromised patients. *Staphylococcus* and *Enterococcus* strains which possess antibiotic-resistant characteristics, and spore-forming bacteria such as *Clostridium difficile*, which produce persistent spores that are inherently resistant to antibiotic attacks from a host’s immune system^20^, have been isolated from aerosolised toilet plumes in previous studies^21–25^. These bacteria and other viruses^5,26^ which have been isolated from toilet plume in healthcare settings, have also been found to be resistant to most hospital-grade disinfectants and can persist even after cleaning^16,26–29^. Thus, these findings reveal the limitations of conventional cleaning methods when combating toilet plume-related pathogens, emphasising the need for increased attention in developing stringent indoor air cleaning and decontamination technologies to address this resistant source of contamination.

Toilet plume bioaerosol sampling and risk assessment studies^5,23,24,30–34^ have suggested interventions such as adequate ventilation, closing the toilet lid and using ultraviolet C (UVC) devices to reduce exposure to bioaerosols via inhalation or contact with fomites. However, none of these studies comprehensively examined the contributing factors that increase the risk of exposure to these bioaerosols before suggesting recommended interventions. Furthermore, the state of the toilet lid and ventilation have been identified as significant factors in reducing exposure to bioaerosols released after toilet flushing and have been investigated under various controlled scenarios^23,24,30^. However, previous investigations have prioritised these factors without providing a clear justification for the number of experiments conducted to determine their significance. Besides, the basis for preferentially selecting and prioritising these factors for investigations seems arbitrary. Additionally, the effect of ventilation and the state of the toilet lid were evaluated independently without considering other possible confounding effects of other factors that could contribute to an increased risk of exposure to bioaerosols. Thus, to develop practical, holistic interventions to minimise exposure to bioaerosols released during toilet flushing, it is imperative to first understand the interactions among the factors contributing to these bioaerosol concentrations. This could be achieved by deploying a Design of Experiments (DoE) methodology.

Design of Experiments (DoE) is a robust and widely used methodology based on statistical principles and used for conducting scientific studies and experiments. Unlike traditional one-factor-at-a-time studies, DoE allows the systematic exploration of multiple factors and their interactions concurrently^35^. This approach uncovers complex relationships and prevents oversimplifications in sequential or isolated experiments. By carefully selecting experimental conditions and sample sizes, DoE maximises the information obtained from each experiment, reducing the cost, effort, time, and other resources involved. Experimental data can then be analysed to develop mathematical models that describe the relationship between the factors studied and desired response^36^. Notably, the statistical foundation of DoE enhances the credibility and validity of the research findings since the methodology is scientifically rigorous, proven, robust and reliable. A systematic review^37^ on toilet plume bioaerosol concentrations from diverse studies revealed discrepancies and substantial variations in magnitude, experimental conditions, and sampling methods, even among similar pathogens. Consequently, replicating and advancing previous research to enhance understanding of the disease transmission risks associated with toilet plume bioaerosols has proven challenging. DoE provides a structured and documented framework for experimental design, execution, and analysis. This enhances the reproducibility of the study, enabling other researchers to validate the results or build upon the findings^35,36^. For the first time, DoE is used in this study and research space to examine the factors that heighten the risk of exposure to bioaerosols following toilet flushing. It also aims to develop an empirical model for predicting the concentration of these bioaerosols. In this study, the term “exposure-relevant bioaerosol concentration” has been used to represent the concentration of potentially infectious bioaerosols that individuals may encounter after toilet flushing, whether through inhalation or fomite exposure. This study also considers how toilet flushing influences the pre-existing relative humidity levels in a toilet environment to affect the indoor air quality, and ultimately, how relative humidity impacts the exposure-relevant bioaerosol concentration, with potential implications for public health. Humidity can influence the survival, dispersion, and transmission potential of bioaerosols. Specifically, high relative humidity has been shown to support the persistence and viability of many pathogens in aerosolized forms, potentially extending their survival time in the air and increasing the risk of inhaled and fomite-mediated infections^38,39^. On the contrary, extremely low humidity levels can also affect bioaerosol dynamics by facilitating the desiccation and aerosolization of pathogens, which may affect their infectivity^38,40^.

With the increase in urbanisation and global focus on achieving Sustainable Development Goals (SDGs) such as Good Health and Well-being (SDG 3), Clean Water and Sanitation (SDG 6), and Sustainable Cities and Communities (SDG 11), there has been a significant rise in the number of public toilets. Consequently, communal/shared public toilets will probably continue to serve as a crucial indicator of adequate, safe access to affordable housing and basic healthcare services, safely managed sanitation, and other relevant environmental impact and public health assessment. To perform effective exposure and risk assessments of toilet plume bioaerosols, understanding key exposure factors is essential. This study quantifies bioaerosol exposure post-toilet flushing and enhances the understanding of toilet plume bioaerosol exposure pathways for risk assessment. The practical implications of this study extend to policymakers, facility managers and public health authorities in healthcare and hospitality (lodging, food and drink services, meetings and event planning, entertainment and recreation, travel, and tourism), aiding policy development and public health protection. Ultimately, this will help advance progress towards the SDGs.

## Methods

### Description of purpose-built indoor toilet used in this study

**Figure 1:**
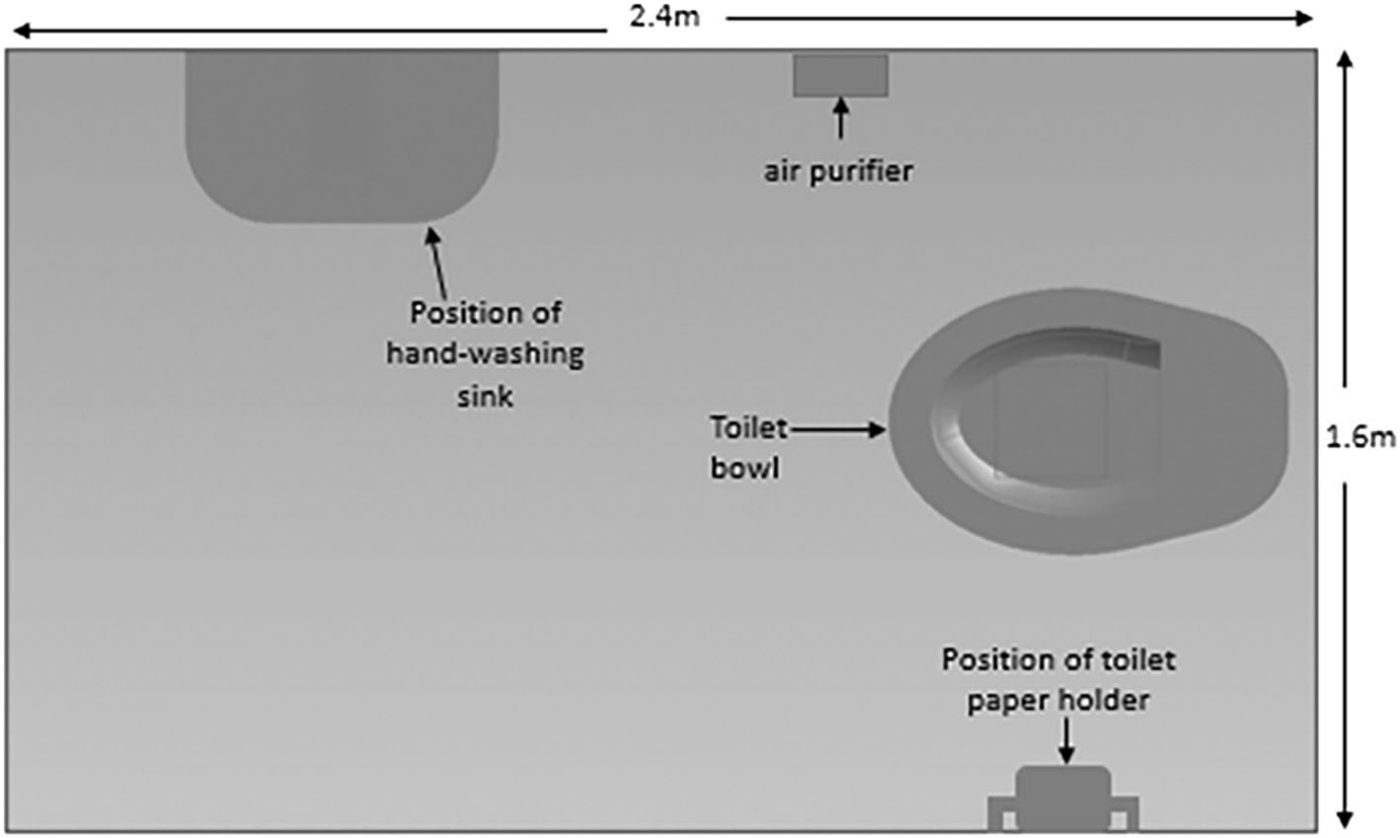
The layout of the specially designed toilet cubicle used in the study.

**Figure 2:**
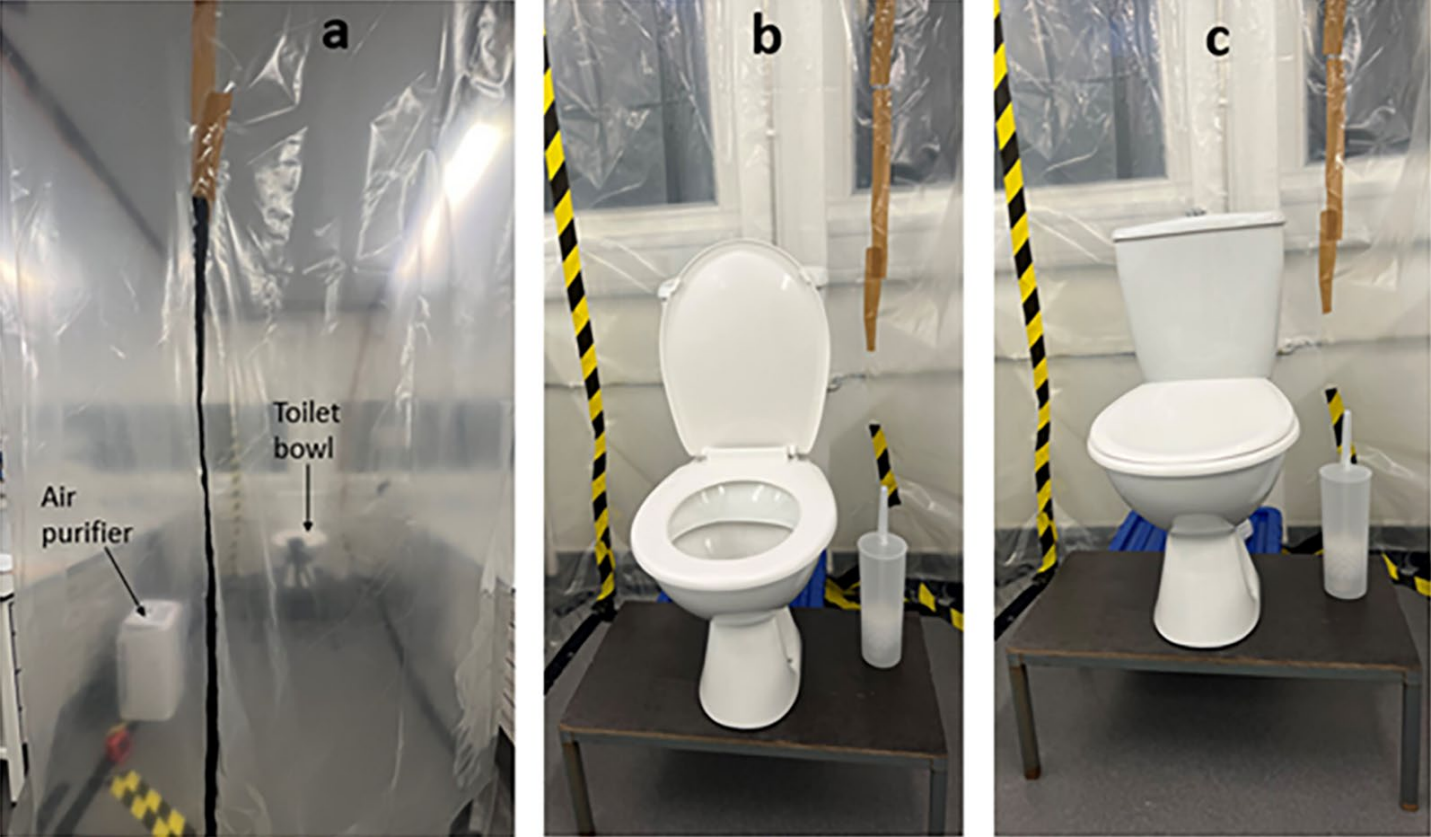
Digital image of the purpose-built toilet cubicle used for experiments. (**a**) Shows the cubicle enclosed with polythene material, with a zip to seal cubicle’s entrance during flushing and sampling experiments. (**b**) Shows the toilet bowl and cistern with lid open. (**c**) Shows toilet bowl with lid closed.

An indoor toilet cubicle (see Figs. 1 and 2) was specially constructed in a microbiological laboratory, to replicate the appearance of a toilet commonly found in healthcare or hospitality environments. The dimensions of the toilet cubicle (2.4 m by 1.6 m and height of 3 m) were informed by the standard requirements specified in BS 6465-1^41^ and Approved Document F of the Building Regulations in the UK^42^ for public toilets found in schools, hospitals, and other commercial and public buildings. A 6-L dual flush toilet (Screwfix, model number SXPTP0056) with a flushing duration of four seconds was installed to simulate a functional toilet unit. The toilet environment was ventilated using a mechanical extraction ventilation system (Vent-Axia, model number W161510) with an extraction rate of 270–395 m^3^/h and an air purifier (Vent-Axia, model number 496611), which provided high-efficiency particulate air (HEPA)-filtered air supply at a clean air delivery rate of 260 m^3^/h. As shown in Fig. 2, the air purifier was positioned to simulate a toilet cubicle's sidewall, reflecting findings from previous studies that sidewall fans offer optimal aerosol removal efficiency^33^. While the utilization of air purifiers in public toilets is not a prevalent practice, previous studies^43,44^ support their application in various indoor environments to effectively mitigate bioaerosols and airborne contaminants. In this study, the inclusion of an air purifier serves the purpose of exploring potential outcomes if such technology were integrated into public toilet ventilation systems. This experimental approach provides valuable insights and has the potential to guide future innovations in the design and enhancement of ventilation systems within public toilets.

The decision to exclude windows and rely on mechanical ventilation equipped with an air purifier was based on a previous study^24^ that demonstrated the superior performance of mechanical ventilation in eliminating bioaerosols. Furthermore, adhering to British standards, windows in public toilets primarily serve the purpose of providing light rather than ensuring proper ventilation. These windows are typically positioned high on the toilet walls to address privacy and security concerns, allowing individuals to feel safe using the facilities. Moreover, using open windows to ensure adequate ventilation is impractical in the study setting, which takes place in a biological laboratory environment and partly due to enclosing the specially designed toilet cubicle with polyethene material. Thus, the exclusion of windows ensures that this study’s findings would be applicable to a wide range of indoor environments, including those where natural ventilation is limited or not feasible. In this study, jet air dryers were excluded, and the cubicle was equipped with paper towels for hand drying, to passively isolate factors that elevate bioaerosol exposure risk post-toilet flushing without the interference of room air currents. Jet air dryers are characterised by powerful airflow and are known to augment the dispersion of bioaerosols and the transfer of bacteria from hands into the toilet environment^15^. Including them in the study would make it impossible to measure the net bioaerosols caused by toilet flushing accurately. This is because the measured bioaerosols would consist of contributions from both toilet flushing and the jet dryer. By utilising paper towels for hand-drying, this study ensures an unbiased evaluation of the factors and minimises potential errors in the modelling process.

### Preparation of non-toxigenic inoculum

This study used a non-toxigenic strain of *Clostridium difficile* (NCTC 13574, National Collection of Type Cultures, UK) as a surrogate for human pathogens. It was used to create spore suspensions to resemble the appearance of diarrheal stool, as *C. difficile* infection is often associated with diarrhoea symptoms. *C. difficile* is a leading cause of antibiotic-associated gastroenteritis and colitis in healthcare settings^20,45^. Aside its antimicrobial resistance, the bacteria is also known for the production of airborne spores that are resilient to heat, standard cleaning and disinfection practices and can persist on surfaces for extended periods^46,47^. *C. difficile*’s presence in toilet plume has been documented in several studies examining toilet plumes^22,48^ so its capability for aerosolization and transmission via the faecal–oral route makes it a critical pathogen of interest in studies of disease transmission via bioaerosols. Furthermore, the non-toxigenic form of *C. difficile* used in this study aligns with its established use as a surrogate in previous assessments of toilet plume-associated risks^22,49^. Preparation of the spore suspensions was based on the approach described by Best et al.^23^ in their previous study on airborne *C. difficile.* The freeze-dried bacteria were rehydrated with nutrient broth, cultured on selective *C. difficile* agar plates (Thermo Scientific™ Brazier's *Clostridium difficile* Selective Agar), and incubated anaerobically at 37 °C for 10 days. During the incubation, the selective agar, containing specific antibiotics and reagents, effectively inhibits the growth of other organisms while promoting the fluorescence of *C. difficile* colonies under UV light. The 10-day incubation period was chosen to let the bacteria deplete all available nutrients, leading to sporulation.

**Figure 3:**
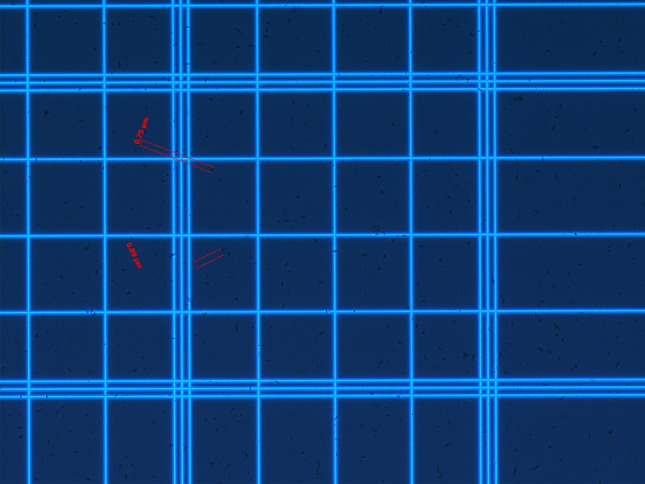
Enumeration of *C. difficile* spores using a hemocytometer under a microscope. This image illustrates the counting of *C. difficile* spores, highlighting their distribution and concentration after being stained with Malachite green solution. Each grid of the hemocytometer is used to systematically quantify the spores. The text in red shows the length of spores.

After incubation, all visible growth from each plate was removed and resuspended in 1 ml of sterile saline. An equal amount of absolute ethanol was subsequently added, and the suspension was left at room temperature for one hour to kill vegetative bacteria. The resulting suspensions were centrifuged at 3000*g* for 15 min, resuspended in 1 ml of sterile water and refrigerated at 4 °C until use. The initial spore suspension was quantified using a hemocytometer under a microscope (see Fig. 3), resulting in a final spore concentration of 10^9^ spores/ml. To determine the total colony forming units of *C. difficile* in the final suspensions for inoculating the toilet, the initial spore suspension was repeatedly diluted. Then, 1 ml of each diluted suspension, was placed onto selective *C. difficile* agar plates. The plates were subsequently incubated anaerobically at 37 °C for 48 h. Once the incubation was complete, the number of visible colonies was counted, and the obtained concentrations were averaged to yield a final suspension concentration of 10^7^ CFU/ml, which were later used to inoculate the toilet in the flushing experiments.

### Design of experiments (DoE)

#### DoE screening design experiments

Previous studies have identified several factors that contribute to the exposure-relevant bioaerosol concentration. These include the concentration of pathogens shed into the toilet bowl, the time elapsed after flushing, the horizontal distance from the toilet bowl, the state of the toilet lid and the presence of a ventilation^4,23,24,30,32^. The collective findings from these studies consistently indicate that the highest concentration of potentially infectious bioaerosols is generated from flushing contaminated toilets immediately after the flush and in the area closest to the toilet bowl. Afterwards, the concentration rapidly diminishes over time, with mechanical ventilation and at greater distances from the source. However, it is important to note that bioaerosols generated can remain in the toilet environment for up to 60 min after flushing, indicating the possibility of lingering contamination^30^.

To achieve the first goal of this study, which is to determine the main factors affecting exposure-relevant bioaerosol levels, a randomised main effects screening design with 12 runs (see Table 1) was used to evaluate the significance of the factors mentioned above suspected to impact exposure-relevant bioaerosol concentration.

**Table 1:** Factors and their levels used in screening design.

Factor	Role	Values	
		Lower limit	Upper limit
Inoculated bacterial concentration (CFU/m^3^)	Continuous	0	10^7^
Time elapsed after flushing (min)	Continuous	0	60
Lateral distance (m)	Continuous	0	1
Mechanical ventilation	Categorical	Off	On
State of lid	Categorical	Closed	Open

Main effects DoE screening experimental designs provide an efficient approach to explore many factors, such as listed, and delineate the most statistically significant factors impacting a response. Once identified via the screening experiments, subsequent experiments can be designed to investigate further these statistically significant factors' main and interactive effects. This streamlined approach reduces the overall number of experiments required, resulting in significant cost and time savings^35,36^. The selection of the levels (i.e., numerical range) of the factors used in the screening design was primarily based on the literature^1,4,13,23–25,50^ and preliminary experimental trials.

While hand hygiene plays a crucial role in infection prevention, it was not included as a study factor due to literature not identifying it as influential on exposure to bioaerosols from toilet plumes post-flushing. Additionally, the decision to exclude hand-washing from our experimental model was based on the objective to establish a baseline understanding of bioaerosol exposure associated with flushing alone, thereby simplifying the model for clarity and specificity in initial investigations.

#### DoE full factorial design experiments

After the screening experiment, a DoE full factorial design was employed as a second step to investigate further the effects and interactions of the most significant factors in the screening design. Full factorial designs are mainly used to examine the effect of all possible combinations of factors and levels on a response by studying the effect of each factor on the response and the effects of interactions between factors on the response^35,36^. Full factorial designs are flexible and can handle complex experimental designs that accommodate continuous, discrete and categorial factors. Four of the five factors examined in the screening design were statistically significant (see section “Conclusion”, “Results”). These factors were incorporated into a 3 × 2 × 2 × 2 full factorial design, as outlined in Table 2, to investigate further these statistically significant factors' main effects and interactions on the exposure-relevant bioaerosol concentrations.

**Table 2:** Factors and their levels used in the full factorial design.

Factor	Role	Number of levels	Values		
			Lower limit	Mid limit	Upper limit
Inoculated bacterial concentration (*C*)	Continuous	3	0	10^6^	10^7^
Time elapsed after flushing (*t*)	Continuous	2	0	N/A	60
Lateral distance (*d*)	Continuous	2	0	N/A	1
Mechanical ventilation (*v*)	Categorical	2	Off	N/A	On

For the purposes of the experimental design and statistical analysis, the continuous variables were discretized into 2 or 3 levels to facilitate a full factorial design approach. The selection of 2 or 3 levels for each factor was informed by statistical principles to ensure sufficient power to detect significant effects and interactions among the factors. This approach aligns with standard practices in experimental design, especially in preliminary studies aiming to identify significant factors for further investigation. Thus, the inoculated bacterial concentration was varied at low and high levels, reflecting a realistic range of concentrations that might be encountered in real-world scenarios. The time elapsed after flushing was chosen to capture immediate (short-term) and delayed (long-term) effects on bioaerosol dispersion**.** The timing chosen for bioaerosol sampling was informed by prior studies indicating significant variability in bioaerosol concentrations immediately following toilet flushing, with a general trend of concentration decrease over time^24^. Sampling at these specific intervals allowed the capturing of the peak bioaerosol concentration and observation of the decline pattern, which is critical for understanding exposure risks and the effectiveness of ventilation in reducing bioaerosol presence. Furthermore, the lateral distance from the toilet was selected to assess the influence of proximity on exposure risk. This allowed the systematic exploration of the impact of these factors and their interactions within the constraints of the experimental setup.

### Air sampling procedure and experimental analyses of bioaerosols generated

Bioaerosol sampling in this study was exclusively conducted by the first author, who was trained in bioaerosol collection techniques to ensure consistency and reliability of the sampling process. Sampling was done using a 400-hole Micro Bio MB1 Bioaerosol Sampler (Cantium Scientific, United Kingdom), with a flow rate of 100 L per minute which featured a delay timer, enabling precise timing for sample collection.

The MB1 bioaerosol sampler was placed directly in front of the toilet bowl, at a height of 0.8 m above the floor, to align with the average adult's sitting posture during toilet use^24^. Before each experimental run, the toilet bowl, and surrounding surfaces were cleaned with hospital-grade bleach disinfectant (5% sodium hypochlorite) and flushed five times to eliminate residual disinfectant and bacteria, as confirmed by preliminary experiments. The bioaerosol sampler was also wiped down with hospital-grade bleach disinfectant before the start of each run. Additionally, regular calibration checks were performed before each set of experiments to maintain measurement accuracy of the sampler. To prevent the contamination of subsequent experimental runs with background bioaerosols from earlier experiments, the air purifier was activated to cleanse the air for an hour after each run, prior to the commencement of each new experimental run.

A new selective *C. difficile* agar plate was positioned within the sampler for each experimental run, with the first author setting the investigated factors to their predetermined levels. This included the addition of a known concentration of *C. difficile* spores to the toilet bowl using a syringe, mounting the sampler directly in front of the toilet bowl at a height of 0.8 m and a distance of either 0 or 1 m from the centre of the toilet bowl, opening or closing the toilet lid, and turning the mechanical ventilation system on or off. With the experimental conditions set, the toilet was flushed, and the first author immediately exited and zipped the cubicle’s entrance. The sampler's delay start feature facilitated immediate sampling of 100 L of air post-flush for runs designated with a time of 0 min. For runs scheduled at 1 h post-flush, the sampler activated one hour after flush, following which the first author re-entered to collect the agar plate for microbial analysis. Air sampling for bioaerosols was done once after flush, for each experimental run. Since this study's primary objective was to examine bioaerosol dispersion dynamics post-toilet flushing, specifically focusing on the factors directly related to the flushing event, the experimental design was structured to minimize variables not directly pertinent to the flushing process, including the detailed personal characteristics of individuals using the toilet. Thus, to ensure consistency across experimental runs and control for variables related to human presence, no individuals were present in the toilet cubicle during the bioaerosol sampling following the toilet flush. The experimental setup maintained a zipped entrance to the toilet cubicle throughout each run, replicating the common scenario in both public and private restrooms where toilets are flushed with doors closed, maintaining privacy and a controlled environment to limit external airflow and bioaerosol movement influences. Once the bioaerosol sampling was done, all recovered agar plates were labelled and incubated anaerobically at 37 °C for 48 h. After the incubation period, colonies were counted using a colony counter. To obtain the actual number of colonies and account for biases in bioaerosol collection, the post-incubation counts were adjusted using the positive-hole method for a 400-hole sampler^51,52^ (1).

1 $${{n}_{c}= {n}_{f}\left[\frac{1.075}{1.052- \frac{{n}_{f}}{{n}_{h}}}\right]}^{0.483}$$

where n_h_ is the number of holes on the sampling head, n_f_ is the number of counted colonies, and n_c_ is the corrected count.

The corrected counts were utilized to calculate the resulting exposure-relevant bioaerosol concentration (*BC*) in CFU/m^3^ as outlined in Eq. (2)^52^.

2 $$BC=1000\frac{{n}_{c}}{{V}_{s}}$$

where V_s_ is the volume of air sampled in litres.

The data gathered from the analysis of plates recovered from the experimental runs consisted of colony counts in CFU. These counts were utilised to calculate the bioaerosol concentration relevant to exposure for the screening and full factorial experiments. This study investigated the environmental impact of flushing an artificially contaminated toilet, emphasizing environmental and microbiological aspects rather than human experimentation. The study did not examine or collect data related to the effects on human subjects, aside from their involvement in flushing the toilet. A risk assessment was conducted and approved for this study, on the basis of not collecting data related to the study’s effects on human subjects and the use of non-toxigenic *C. difficile* spores to minimize health risks. Additionally, the experiments were carried out in an isolated Class 2 biological laboratory, where entry was restricted exclusively to the human operator conducting the toilet flushing experiments. This operator was equipped with protective gear to ensure added safety during the experiments.

### Monitoring of relative humidity (RH)

The RH during each experimental run was monitored per the WHO indoor air quality guidelines^53^ using the RS PRO DT802D air quality monitor and data logger (RS Components Ltd., United Kingdom), capable of detecting RH levels ranging from 0 to 90% with an accuracy of ± 5% RH and a precision of 0.1%. The device was placed at a height of 1 m above the floor, at the centre of the toilet cubicle to ensure that the measurements reflected a well-mixed air sample, representative of the cubicle's general air quality post-flushing. The interplay of how RH and bioaerosol levels affect the air quality in the toilet environment was examined using randomised continuous flushing. Thirteen randomised continuous flushing experiments were conducted, and significant changes were observed in the RH concentrations post-toilet flushing levels.

### Bioaerosol concentration modelling

Each run was replicated three times to increase reliability and reduce the possibility of variability. Thus, there were a total of 108 randomised experimental runs. In the full factorial experimental setup, the influence of four primary factors on the concentration of bioaerosols following toilet flushing were directly investigated. This included the initial concentration of *C. difficile* spores added to the toilet bowl prior to flushing (C), the duration after flushing during which bioaerosol samples were collected (t), the distance at which bioaerosol samples were taken from the toilet (d) and the presence and efficiency of mechanical ventilation in reducing bioaerosol concentrations (v). An empirical model was developed to predict exposure-relevant bioaerosol concentration, incorporating both the directly tested factors and their interactions based on experimental data. Specifically, the interactions between lateral distance and time after flushing (d × t), inoculated bacterial concentration and time after flushing (C × t), and inoculated bacterial concentration and lateral distance (C × d), were included to capture the combined effects of these variables on bioaerosol concentration. To determine the relationship between the concentration of exposure-relevant bioaerosols and the factors involved, the results of the experiments were analysed using regression analysis and fit into a second-order polynomial equation (see Eq. (3)).

3 $$Y={\beta }_{0}+{\beta }_{1}{x}_{1}+{\beta }_{2}{x}_{2}+{\beta }_{3}{x}_{3}+{\beta }_{4}{x}_{4}+{\beta }_{11}{x}_{1}^{2}+{\beta }_{22}{x}_{2}^{2}+{\beta }_{33}{x}_{3}^{2}+{\beta }_{44}{x}_{4}^{2}+{\beta }_{12}{x}_{1}{x}_{2}+{\beta }_{13}{x}_{1}{x}_{3}+{\beta }_{14}{x}_{1}{x}_{4}+{\beta }_{23}{x}_{2}{x}_{3}+{\beta }_{24}{x}_{2}{x}_{4}+{\beta }_{34}{x}_{3}{x}_{4}+\varepsilon $$

where Y = predicted exposure-relevant bioaerosol concentration (*BC*), β_0_ = intercept or constant term, x_1_, x_2,_ x_3_ and x_4_ = *C, t, d* and *v* respectively, β_1_, β_2_, β_3_ and β_4_ = linear coefficients for *C, t, d* and* v* respectively, β_11_, β_22_, β_33_ and β_44_ = quadratic coefficients for *C, t, d* and* v* respectively, β_12_, β_13_, β_14_, β_23_, β_24_ and β_34_ = interaction coefficients between *C, t, d* and* v, *ε = random experimental error term.

To compare the means and assess the statistical significance of the obtained model and terms, Analysis of Variance (ANOVA) and p-value were used. The predetermined significance level was 0.05, meaning values equal to or below this threshold were considered statistically significant. The precision and reliability of the model fitting for predicting the exposure-relevant bioaerosol concentrations were assessed using R^2^ and adjusted R^2^. DoE, statistical and graphical analyses were done using JMP statistical software.

### Additional information

This study examined the environmental impact of flushing an artificially contaminated toilet without directly affecting or gathering data from human subjects beyond the act of flushing. Thus, it aligns more with environmental or microbiological research than human experimentation. The involvement of a human operator (first author) to flush the toilet, is aimed at mimicking real-life conditions accurately within a controlled setting. Except for the action of flushing, no other human activities were conducted in the toilet cubicle during the sampling to isolate the effect of flushing on bioaerosol dispersion. The involvement of a human operator in the flushing process was conducted with strict adherence to safety guidelines and a risk assessment from Loughborough University which was approved on the basis of no data being collected from human subjects, wearing protective gear at all times and the use of non-toxigenic *Clostridium difficile* to reduce health risks. The approved risk assessment document is available upon request. These precautions ensured the study adhered to health and safety standards while accurately replicating toilet flushing dynamics in a controlled environment.

## Results

### Main effects DoE screening analysis of all factors

According to the results of the screening experiments (see Supplementary Table S1) the bacterial concentration (*C*) that was inoculated, the time that elapsed after flushing (*t*), the lateral distance from the toilet bowl (*d*), and the presence of mechanical ventilation (*v*) were all statistically significant factors in reducing the risk of exposure to potentially higher concentrations of infectious bioaerosols during toilet flushing. The screening analysis model yielded an R-squared value of 0.96 and a p-value of 0.0003.

### DoE Full factorial analysis

#### Analysis of the main effects of contributing factors

Results from the DoE full factorial experiments (see Supplementary Table S2) showed that the time elapsed after flushing (p < 0.0001), the concentration of inoculated bacteria (p < 0.0001), and the lateral distance from the centre of the toilet bowl (p < 0.0001) all had the most significant main effects. Whilst both the time elapsed after flushing and the lateral distance from the centre of the toilet bowl had a negative linear effect on the exposure-relevant bioaerosol concentration, the inoculated bacterial concentration produced a positive linear effect. Furthermore, the mechanical ventilation system had a significant main effect (p = 0.0114), although its impact was not as notable as that of *C, t* and *d.*

#### Analysis of Interactions among contributing factors

The interaction terms, lateral distance × time elapsed after flushing (*d* × *t*; p < 0.0001), inoculated bacterial concentration × time elapsed after flushing (*C* × *t*; p = 0.001), and inoculated bacterial concentration × lateral distance (*C* × *d*; p = 0.004), were also found to be statistically significant, revealing the interplay and intertwined combined impacts of contributing factors on the exposure-relevant bioaerosol concentration. This is critical to enable accurate empirical modelling and determination of exposure-relevant bioaerosol concentrations.

##### Analysis of *C* × *d* interactive effect

**Figure 4:**
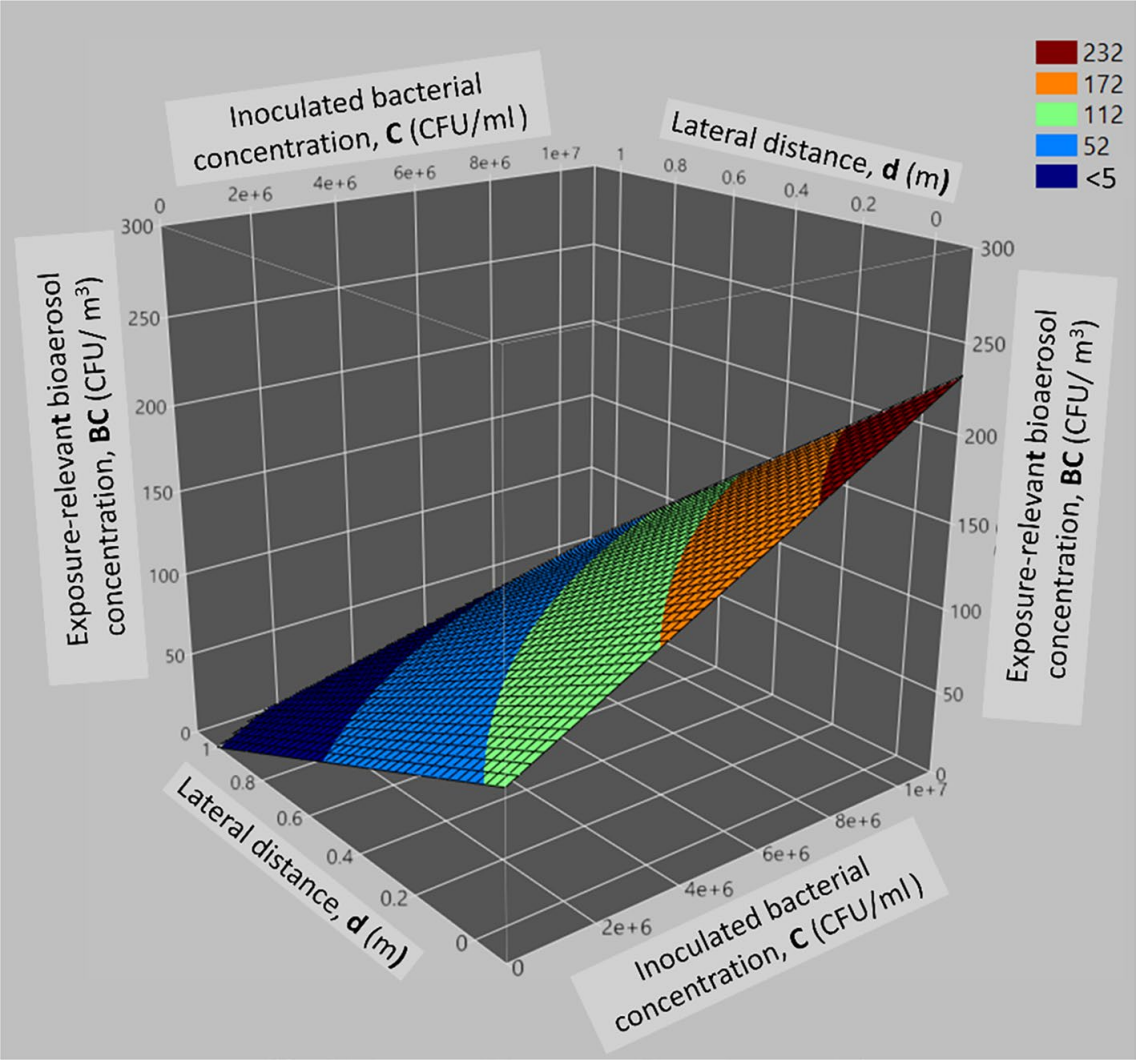
Surface plot illustrating the interactive effect of *C* and *d* on the exposure-relevant bioaerosol concentration.

The interaction term, *C* × *d*, revealed a notable dependence of the exposure-relevant bioaerosol concentration on both the inoculated bacterial concentration and lateral distance from the toilet bowl. In Fig. 4, it is observed that as *C* increases and *d* decreases, *BC* increases. At the lowest *d*, a rise in *C* correlates with a steady BC increase from 0 CFU/m^3^ to approximately 232 CFU/m^3^. However, when *C* is minimal, increasing *d* has no prominent effect on *BC*. With increasing *C* and *d*, *BC* rises from 0 CFU/m^3^ to about 52 CFU/m^3^ when C reaches 10^7^ CFU/ml and *d* extends to 0.1 m. As both *C* and *d* continue to increase, BC stabilizes at 52 CFU/m^3^ until *d* reaches around 0.8 m. Beyond 0.8 m, the influence of increasing *C* on *BC* gradually diminishes, fluctuating between 0 CFU/m^3^ and 5 CFU/m^3^ at the farthest *d*.

##### Analysis of *C* × *t* interactive effect

**Figure 5:**
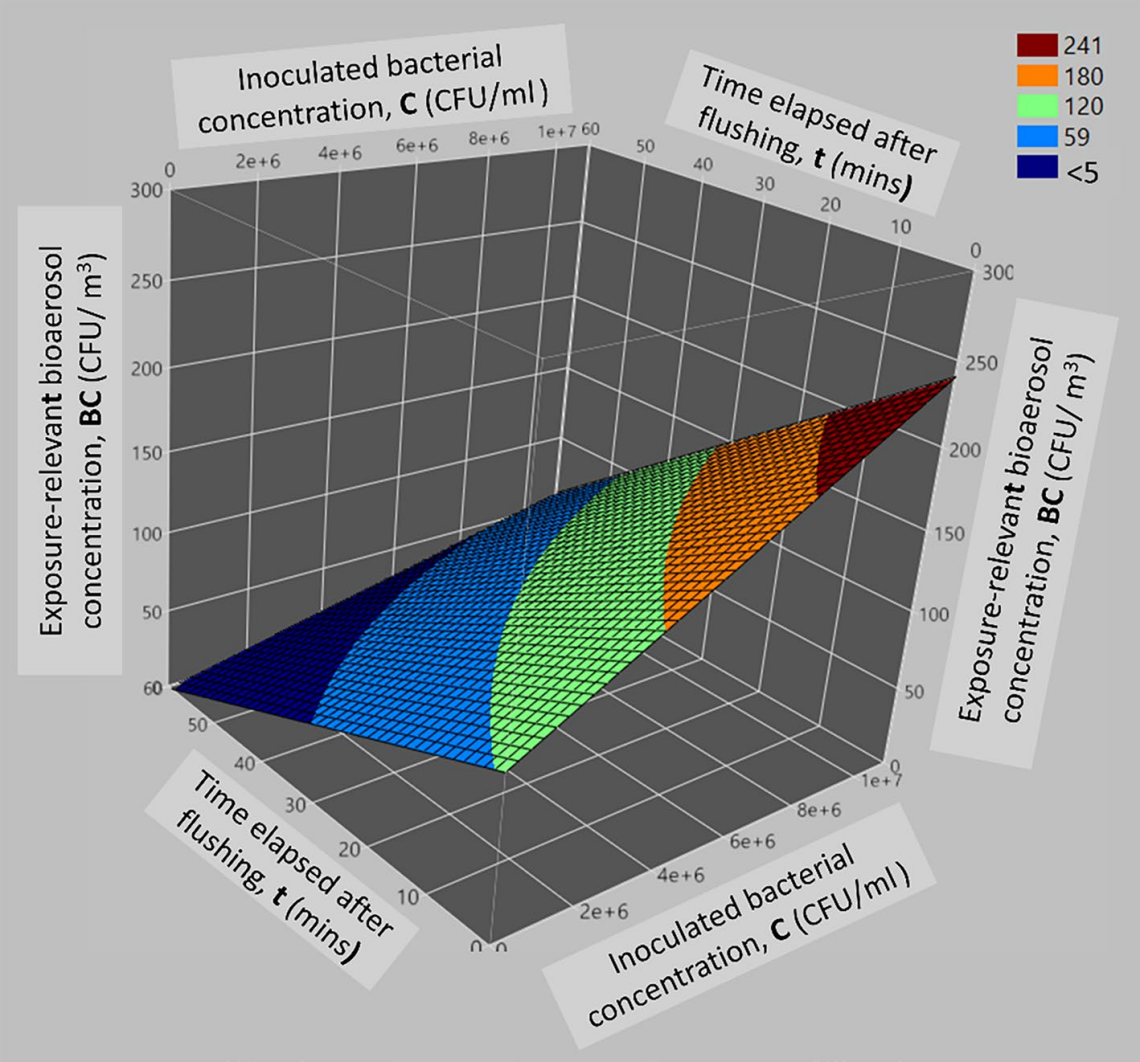
Surface plot illustrating the interactive effect of *C* and *t* on the exposure-relevant bioaerosol concentration.

The interaction term *C* × *t* reveals that higher concentrations of inoculated bacteria may exert varying effects on exposure-relevant bioaerosol concentration at different time intervals. As depicted in Fig. 5, *BC* rises with increased *C* and decreased *t*. At the lowest *t*, increasing *C* leads to a continuous rise in *BC* from 0 CFU/m^3^ to around 241 CFU/m^3^. With rising *C* and *t*, *BC* increases from 0 CFU/m^3^ to about 59 CFU/m^3^ when *C* hits 5 × 10^6^ CFU/ml and *t* extends to 10 min. *BC* stabilizes at 77 CFU/m^3^ as *C* and *t* continue to increase up to around 40 min. Beyond 40 min, the impact of increasing *C* on *BC* gradually diminishes from 59 to 0 CFU/m^3^ at the highest *t*.

##### Analysis of *d* × *t* interactive effect

**Figure 6:**
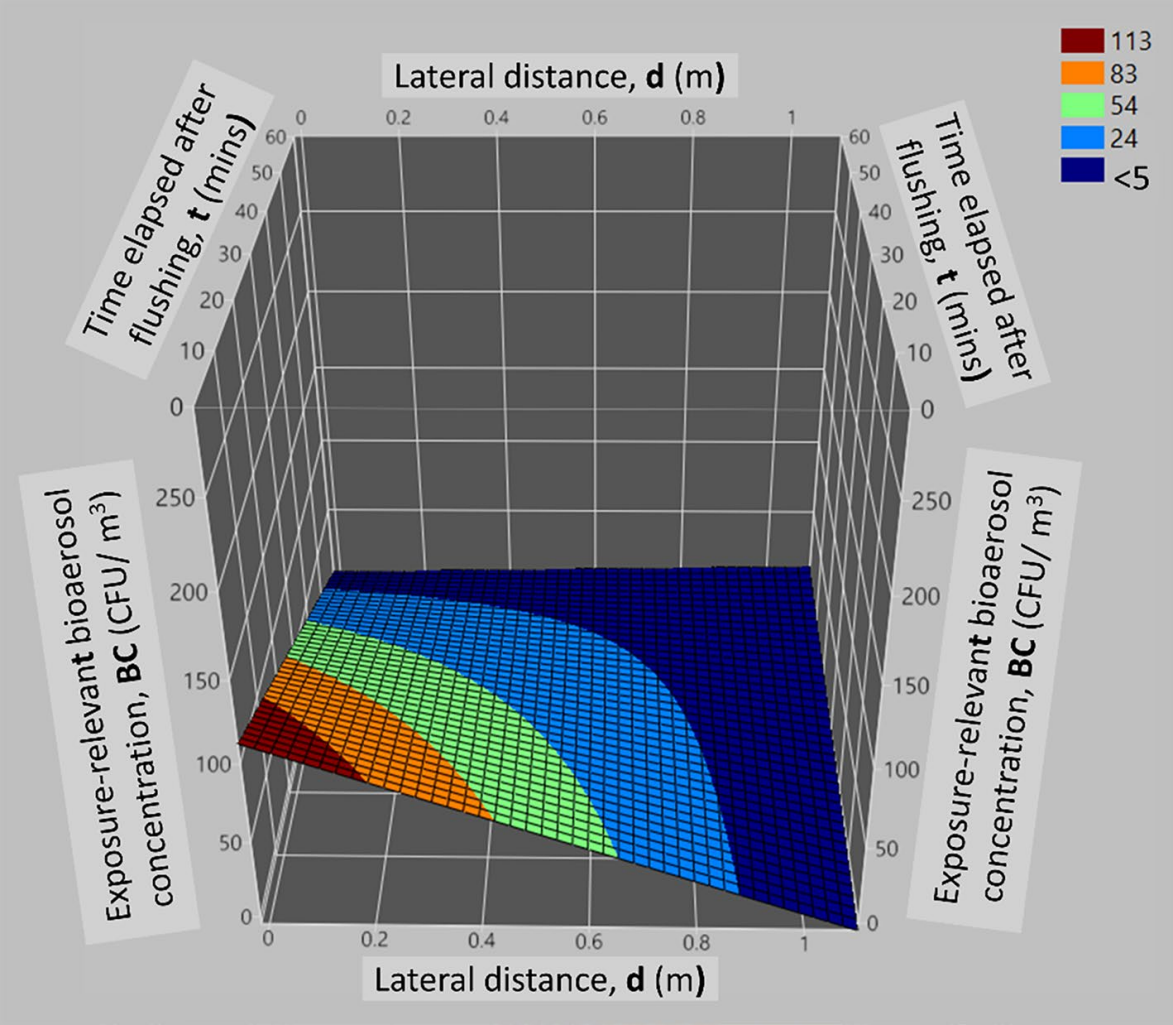
Surface plot illustrating the interactive effect of *d* and *t* on the exposure-relevant bioaerosol concentration.

The *d* × *t* interaction revealed an inverse relationship between the variables, where greater lateral distance from the flushing source and longer time post-flush contributed to reduced bioaerosol concentrations, while reduced later distance and shorter time post-flush corresponded to elevated bioaerosol levels. This is evident in Fig. 6, where a simultaneous increase in both *d* and *t* results in a decrease in *BC*, while conversely, a simultaneous decrease in both *d* and *t* leads to an increase in *BC*.

### The impact of toilet flushing on the air quality in toilet environment

After conducting thirteen randomised continuous flushing experiments, significant changes were observed in the relative humidity concentrations post-toilet flushing levels. During the monitoring period, it was noticed that the relative humidity (see Supplementary Fig. S1) increased immediately after every flush. After this, the levels gradually decreased after about 10 min of initiating the flush.

### Exposure-relevant bioaerosol concentration prediction model

Equation (4) presents the simplified empirically derived prediction model for exposure-relevant bioaerosol concentration determination.

4 $$BC=82.39+1.11{e}^{-5}C-87.96d-1.44t-0.61{e}^{-5}Cd-0.01{e}^{-5}Ct+1.83dt+10.28$$

The prediction model was highly significant (p < 0.0001). The R^2^ and adjusted R^2^ were 0.72 and 0.68, respectively.

## Discussion

The screening design results revealed that among the studied factors only *C*, *d*, *t* and *v* were significant in influencing the exposure-relevant bioaerosol concentration. Higher concentrations of pathogens in the toilet bowl can result in more pathogens being released into the air, increasing the microbial load on surfaces and elevating the risk of pathogen exposure through inhalation or surface contact^4,7^. As time elapses after flushing, the exposure-relevant bioaerosol concentration decreases due to natural processes like gravitational settling and dispersion in the surrounding air^1^. Mechanical ventilation systems are vital for air exchange, replacing contaminated air with fresh air, which reduces bioaerosol concentrations. HEPA filters in these systems capture airborne particles, including bioaerosols, effectively^24,43,44^. The distance from the toilet bowl significantly affects bioaerosol concentrations, as bioaerosols have less room to disperse and dilute at shorter distances from the toilet bowl, resulting in higher concentrations. Conversely, these bioaerosols disperse more effectively at greater distances, resulting in lower concentrations. This phenomenon elucidates the observed contamination of surfaces near toilet seats after flushing, as noted in previous studies^4,54,55^. In this study, whether the toilet lid is open or closed did not exert a significant influence on bioaerosol concentration. This suggests that lid positioning alone is not an effective means of reducing bioaerosol levels, implying the involvement of other contributing factors. This finding aligns with the existing literature, which presents conflicting evidence. Some studies report reduced bioaerosol levels when the lid is closed^56,57^, while others contend that gaps between the seat and lid may permit bioaerosol escape^8^. Furthermore, lifting or adjusting the lid during subsequent toilet use can disrupt contained bioaerosols, particularly in toilets with high flushing energies (400 kPa or greater than 50 psi particle-free water supply to the toilet)^1,25^. Screening experiments assess the main effects, neglecting confounding factors, potentially leading to non-linear relationships between *C*, *d*, *t*, or *v* and bioaerosol concentration. Relying solely on screening results is inadequate, as factors like time elapsed post-flush and lateral distance are closely tied to dispersion and dilution affected by ventilation and air currents. To address this, further investigation through a full-factorial design was crucial.

In the full factorial analysis, the observed variations in the magnitude of main effects can be attributed to measurement method sensitivity, with *C*, *t*, and *d* showing more pronounced effects than mechanical ventilation. Nevertheless, the role of mechanical ventilation in reducing bioaerosol levels is vital. Mechanical ventilation’s significance is highlighted by previous research, indicating a twofold decrease in disease burden for infections linked to toilet plume bioaerosols when air exhaust ventilation is active^58^. This is attributed to the fresh air exchange and airflow that mitigate stagnant air and bioaerosol accumulation. In high-traffic public toilets these factors result in elevated exposure-relevant bioaerosol concentrations^24,59^. The less pronounced main effect of mechanical ventilation on the exposure-relevant bioaerosols, in comparison to *C*,* t*, and *d*, also suggests that ventilation alone may not significantly reduce bioaerosol concentrations. The effectiveness of mechanical ventilation is likely influenced by airflow rate, direction, distribution towards the flushing source, and air cleaning processes, elements not investigated in our current study.

The *C* × *d* interaction infers that, individuals positioned nearest to the toilet bowl post-flush face a heightened risk of exposure to potentially elevated bioaerosol concentrations. This finding sheds light on the complex dynamics of bioaerosol dispersion and spatial concentration distribution after toilet flushing, emphasizing the pivotal role of distance in assessing exposure risks and developing effective mitigation strategies. While controlling pathogen concentrations in faeces may be challenging, the broader significance lies in advocating physical distancing measures to reduce exposure to toilet plume bioaerosols. Targeted interventions, considering the interaction between distance and concentration, can be implemented, such as stricter cleaning protocols that prioritise thorough and regular cleaning of surfaces nearest to the toilet bowl. Furthermore, public toilet designs should incorporate easily cleanable, antimicrobial surfaces adjacent to the toilet bowl to minimize exposure. Effective ventilation is crucial for bioaerosol dilution and removal and ventilation systems in toilets can be localised and optimised to effectively capture and remove bioaerosols, especially in areas closer to the toilet bowl.

The *C* × *t* interaction reflects the combined effect of droplet settlement, microorganism viability and bioaerosol dispersion, collectively influencing the exposure-relevant bioaerosol concentrations. Immediately after a toilet is flushed, the initial bioaerosol concentration is notably elevated, but over time, the viability of some microorganisms diminishes significantly. This can be attributed to the turbulence induced by aerosolization during flushing, the efficiency of ventilation, and unfavourable environmental conditions for anaerobic microorganisms like the *C. difficile* used in this study^32,54,55,60^. As time elapses, effective ventilation within the toilet environment aids in the desiccation of suspended bioaerosols, lowering their viability and reducing bioaerosol concentrations. Additionally, air currents within the toilet contribute to the dispersal and dilution of suspended bioaerosols. This aligns with prior research indicating a heightened health risk from toilet plume bioaerosols during the initial 0–15 min after flushing, which becomes tolerable around the 35-min mark^24^. The peak concentration of exposure-relevant bioaerosols typically transpires within the initial 10 min post-flush, as larger droplets settle swiftly^40,61,62^, contaminating surfaces near the toilet bowl. Consequently, the number of airborne droplets contributing to the bioaerosol concentration decreases significantly over time. The interactive effects of *C* × *t* imply the need for targeted interventions during critical time intervals and peak exposure periods post-flush. Timely cleaning and disinfection protocols can reduce the initial bioaerosol concentration released post-flush. Moreover, maintaining residual disinfectant in the toilet flushing water can lower the concentration of pathogens shed into the bowl, thereby reducing infectious bioaerosol generation. The requirement of improved ventilation is another implication of this interactive effect; with adjustable airflow rates to accommodate the changing bioaerosol concentrations and dispersal patterns over time. EU standard EN16798-1 recommends a ventilation rate of 10 L/s/person in isolated spaces or continuous operation of exhaust fans for effective ventilation^54^. This standard can be integrated into ventilation system design for more precise regulation of toilet ventilation to address varying bioaerosol propagation dynamics. While this interactive effect highlights the interplay between bacterial concentration, time, and environmental factors in affecting post-flush bioaerosol concentrations, a comprehensive analysis of the experimental setup is essential to explore specific environmental conditions, bacterial species, and other factors impacting exposure-relevant bioaerosol concentration.

The interaction term, t × d, sheds light on how both time and lateral distance influence the concentration of exposure-relevant bioaerosols. This interaction manifests differently at various lateral distances from the toilet bowl, implying a complex interplay between these variables in the dispersion of bioaerosols in the toilet environment. When a toilet is flushed, bioaerosols are released and disseminated into the surrounding space. Over time, these bioaerosols disperse, moving away from the flushing source. The rate of this dispersion depends on factors like air currents^24,43,44^, turbulence, and physical barriers. Initially, the concentration of bioaerosols is higher at closer lateral distances due to proximity to the flushing source. However, with increasing time elapsed after flushing, the impact of air currents in drying and transporting bioaerosols becomes evident, leading to a decline in their concentration. These findings align with previous research indicating a rapid decrease in bacterial bioaerosols as time passed and sampling locations moved further from the flushing source^37^. The *t* × *d* interaction is more pronounced at shorter distances, where bioaerosols have additional time to scatter and become diluted as they travel longer lateral distances, resulting in a rapid reduction in concentration over time. To mitigate bioaerosol concentrations at shorter distances and hinder their dispersion, higher ventilation rates equipped with localized exhaust systems or high-efficiency air filters capable of swiftly removing bioaerosols are essential. In this study, the main effect of *t* has a more pronounced effect compared to that of *d* and *C*. Notably, after 60 min, the importance of lateral distance and bacterial concentration diminishes.

From the randomised flushing experiments, increased water droplets are introduced, which increases the moisture levels in the toilet environment. This causes the relative humidity levels to rise immediately after flushing. High relative humidity levels prolong the desiccation of bioaerosols^63^, support the persistence and viability of many pathogens in aerosolized forms, potentially prolonging their survival time in the air and heightening the risk of inhalation and surface deposition for infectious disease transmission^38^. Furthermore, high relative humidity levels increase the transfer efficiencies of pathogens from contaminated surfaces (fomites) and this poses a higher risk of fomite-mediated infections^39^. The implications of relative humidity on bioaerosol propagation behaviour and viability of potentially aerosolised pathogens highlight the importance of managing relative humidity within the recommended 40–60% range for human health in indoor environments^64,65^. This can be achieved using dehumidifiers, proper HVAC systems, or ventilation strategies that promote air exchange and moisture control to minimise health risks associated with exposure to potentially pathogenic bioaerosols. The combination of inadequate ventilation and elevated relative humidity levels creates a breeding ground for pathogen-laden bioaerosols, including moulds. Hence, and based on the results of this study, public toilets that experience heavy usage and frequent flushing may pose a greater risk of exposure to potentially harmful bioaerosols.

The R^2^ and adjusted-R^2^ of the simplified empirically derived prediction model for exposure-relevant bioaerosol concentration suggests that a large part of the variation in the bioaerosol concentration was accounted for in the model. The study explored how different factors contribute to the increased risk of exposure to bioaerosols after flushing a toilet. The study focused on the concentration of pathogens shed into the toilet bowl, the distance from the toilet, the time elapsed after flushing, and the mechanical ventilation system in the toilet environment. Although the study revealed important insights, it is important to acknowledge the limitations of the model used to predict the exposure-relevant bioaerosol concentration.

Despite rigorous calibration and standardization of our sampling and analysis methods, inherent variability in bioaerosol sampling and spore enumeration techniques could introduce uncertainty to the measured concentrations of *C. difficile* spores. This variability is a common challenge in bioaerosol research and was mitigated as much as possible through repeated measurements and adherence to established protocols. Additionally, slight fluctuations in humidity and air exchange might have influenced the dispersion and survival of bioaerosols, albeit minimally due to the controlled environment. The empirical model developed to predict exposure-relevant bioaerosol concentration was based on specific experimental conditions and factors. While it provides valuable insights, the model's applicability to different settings, especially those with natural ventilation or varying occupancy patterns, may introduce uncertainty regarding its generalizability. The use of non-toxigenic *C. difficile* spores as a surrogate for pathogenic bioaerosols allows for safe experimentation but also introduces uncertainty regarding the behaviour of other pathogens in similar conditions. The resistance and survivability of different pathogens can vary significantly, potentially affecting the applicability of our findings across different microorganisms.

While the model's fit is adequate given the highly diverse factors involved in bioaerosol generation, there is some lack of fit in the residual data, indicating that the generation of bioaerosols and airflow is highly variable and complex, making it challenging to capture in a single model fully. Therefore, focusing on statistically significant factors, highlighted in literature, that meaningfully contribute to understanding the phenomenon of bioaerosol generation and propagation was more pertinent. This study contributes to a growing body of knowledge on bioaerosol dynamics post-toilet flushing, providing a foundation for future research to build upon and address these uncertainties more comprehensively.

Additionally, while the predictive model may not fit perfectly, it did reveal new patterns, relationships, and areas worthy of further investigation. The analysis conducted in this study did identify statistically significant factors that are crucial for comprehending the heightened risk of bioaerosol exposure. Specifically, pathogen concentration, lateral distance from the toilet bowl, and time elapsed after flushing were significant contributors to bioaerosol exposure risk. These findings are consistent with previous research^4,23,30,58,66^ showing a greater risk of exposure to bioaerosols when there are higher pathogen concentrations, shorter lateral distances, and shorter flushing periods. It is imperative to emphasize that the numerical values presented in this study do not represent standardized thresholds for potentially elevated bioaerosol concentrations. Instead, the significance lies in the discerned trends unveiled by this study.

## Conclusion

This study’s empirical exploration into the propagation of bioaerosols post-toilet flushing has yielded critical insights into the factors most influential in shaping bioaerosol exposure risks. The interaction analyses reveal that bioaerosol concentrations are most significantly affected by the combination of pathogen load and proximity (C × d), with the highest bioaerosol concentrations encountered at the nearest distances and highest bacterial concentrations shed into the toilet bowl. The temporal dynamics of bioaerosol viability (C × t) further highlight the critical window immediately post-flush, where concentrations can surge to the highest, and subsequently diminishing as mechanical ventilation and natural dispersal mechanisms take effect. The relationship between time and lateral distance from the flushing source (d × t) emphasizes the efficacy of distancing and temporal passage in reducing bioaerosol concentrations, highlighting the importance of mechanical ventilation in promoting air quality. Furthermore, this study showed toilet flushing causes temporary humidity fluctuations, highlighting the role of mechanical ventilation in reducing bioaerosol viability and associated health risks by maintaining humidity at safe levels. This study’s findings advocate for the integration of enhanced ventilation systems, strategic facility design, and rigorous cleaning protocols to mitigate bioaerosol exposure. Furthermore, the negligible effect of toilet lid position on bioaerosol levels prompts a re-evaluation of existing recommendations, suggesting a more complex interplay of factors warrants further investigation. By providing a quantitatively rich analysis of bioaerosol dynamics, this study contributes valuable knowledge towards the development of more effective public health strategies in managing bioaerosol risks in communal toilet environments. Future research should extend these findings by incorporating additional variables such as user behaviour and hand-washing, variables related to human presence such as gender, weight, height, attire, duration of toilet use, toilet design, comparative effectiveness of natural versus mechanical ventilation and pathogen characteristics to develop a more holistic understanding of bioaerosol exposure risks and mitigation strategies. Future studies should also build on the findings of this study, to investigate how factors like airflow rate, direction, distribution towards the flushing source, and air cleaning processes influence the effectiveness of mechanical ventilation.

The results of this study support the goals of SDGs 3, 6, and 11 and provide insights for their implementation. By identifying the factors that contribute to exposure to toilet plume bioaerosols, the study can help improve sanitation practices and infrastructure in public areas, thus promoting informed decisions and actions in response to critical environmental health challenges.

### Supplementary Information

Supplementary Information.

## Data Availability

Data processed in this study are included in the article and supplementary information. The raw datasets collected from experiments are available from the corresponding author on reasonable request.
