# Supplementary material for: Exploring toilet plume bioaerosol exposure dynamics in public toilets using a Design of Experiments approach
Source: Sci Rep. 2024 May 9;14:10665. doi: 10.1038/s41598-024-61039-w (PMC11082142; doi:10.1038/s41598-024-61039-w)
Supplement: Supplementary file 1 — Supplementary Information. [file 41598_2024_61039_MOESM1_ESM.pdf]

SUPPLEMENTARY INFORMATION

EXPLORING TOILET PLUME BIOAEROSOL EXPOSURE DYNAMICS IN PUBLIC TOILETS USING A DESIGN OF EXPERIMENTS APPROACH.

Elizabeth N. Paddy*[1], Oluwasola O.D. Afolabi[1], M. Sohail[1],

[1]School of Architecture, Building and Civil Engineering, Loughborough University, Loughborough, Leicestershire, United Kingdom **Corresponding Author**

Contact Information:

Elizabeth N. Paddy

Postal address: School of Architecture, Building and Civil Engineering, Loughborough University, Loughborough, Leicestershire, United Kingdom

Email: e.n.osei@lboro.ac.uk

Supplementary Table S1. Results of DoE screening design experiment

**Factors**	**P- value**
Inoculated bacterial concentration	<0.001
Time elapsed after flushing	0.010
Lateral distance	0.040
Mechanical ventilation	0.004
State of Lid	0.090

Supplementary Table S2. Results of full factorial analysis

**Source**	**Sum of Squares**	**Degree of Freedom**	**Mean square**	**F Ratio**	**p-value (Prob >F)**
**Model**	251399.42	10	25139.90	16.06	<0.0001
C	27664.96	1	27664.96	17.68	<0.0001
t	78380.31	1	78380.31	50.09	<0.0001
d	67290.07	1	67290.07	42.99	<0.0001
v	10646.16	1	10646.16	6.80	0.0114
C* × *t	20225.76	1	20225.76	12.92	0.0006
C* × *d	13825.67	1	13825.67	8.83	0.0042
t* × *d	54450.00	1	54450.00	34.79	<0.0001
C* × *v	4738.58	1	4738.58	3.03	0.0869
t* × *v	2688.89	1	2688.89	1.72	0.1948
t* × *v	2222.22	1	2222.22	1.42	0.2380
**Pure error**	3133.33	48	65.28		
**Lack of fit**	92328.36	13	7102.18	108.80	<0.0001

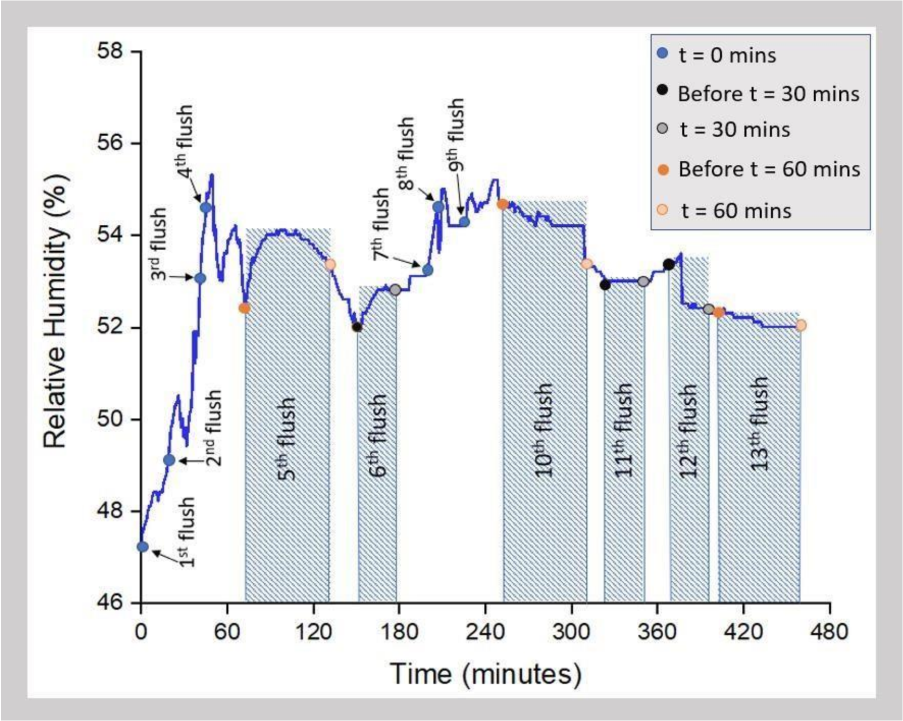

Supplementary Figure S1. Changes in relative humidity levels over time after randomised flushing.
